# From the question how to act in a sustainable manner, back to the question why we act unsustainably

**DOI:** 10.1007/s13280-024-02024-5

**Published:** 2024-04-23

**Authors:** Else Ragni Yttredal, Jörg Löffler, Kenneth M. Tschorn

**Affiliations:** 1 Department of International Business, Norwegian University of Science and Technology (NTNU), PO Box 1517, 6025 Ålesund, Norway; 2 Department of Geography, University of Bonn, Meckenheimer Allee 166, 53115 Bonn, Germany

Comment to: O'Brien, K., R. Carmona, I. Gram-Hanssen, G. Hochachka, L. Sygna, and M. Rosenberg. 2023. Fractal approaches to scaling transformations to sustainability. *Ambio* 52: 1448–1461. 10.1007/s13280-023-01873-w.

In their article, O’Brien et al. (2023) propose a fractal approach to scaling sustainability transformations grounded in "universal values”. This very perspective spurs our eagerness to engage in a discussion about the article. The reason being a critical concern: what if these universal values work against the principles of sustainability? The essence of our commentary revolves around the notion that to comprehensively comprehend the mechanisms underlying sustainability transformations, it is imperative to delve deeper into the foundational factors contributing to the current unsustainable nature of human behaviours.

While there are various approaches and theories in the field of behavioural sciences, some argue that human behaviour can broadly be attributed to four categories: habits, knowledge, opportunities, and values (Brox 1999; Stern 2000). Using this lens, **habits** are behaviour that is repeated regularly and are formed in a complex interplay of psychological, neurological, and environmental factors (See for instance Graybiel 2008 for their neurological basis). Habits play a pivotal role in shaping human behaviour, and while important for sustainable behaviour, we leave habits aside for now. By now it seems evident that **knowledge** alone is insufficient to drive sustainable actions, as the accumulation of decades of research and information (such as reports from the IPCC and IPBES) has ensured that not only researchers but also individuals and policymakers should possess a substantial understanding of the consequences of our unsustainable behaviours. Regarding the aspect of **opportunity**, the global community has so far exhibited reluctance to fundamental transformations of structures that at present facilitate unsustainable behaviour, for example in the field of the economy. Concurrently, our increasing number and advancements in technological and other fields have amplified our capacity to harm the Earth systems (Steffen et al. 2015).

There are, however, many opportunities to act sustainably. It is, therefore, a puzzle why actors simply do not choose sustainable alternatives even when they exist—and actors have the knowledge. This is one of the reasons why we welcome a greater emphasis on diverse aspects of **values** in the discourse on sustainable development and sustainability transformations in other recent articles (Pascual et al. 2023) as well as in O’Brien et al.’s publication (2023). Still, we question the latter’s understanding of the concept of "universal values" as “intrinsic and shared qualities and characteristics that connect humans and nature in an acausal, coherent manner” (O’Brien et al. 2023, p. 1452). O’Brien et al. continue: “Here, acausal describes a connection that is innate and entangled, and coherent refers to forming a whole. As opposed to culturally determined values, universal values ‘transcend religious tenets, norms, and other social diktats’” (O’Brien et al. 2023, p. 1452). Do “universal values” then resemble a fundamental law of nature? Or does the authors’ understanding of universal values have a normative aspect?

Our own empirically based research shows that actors tend to prioritise social issues compared to the economic and biophysical aspects related to sustainability (Yttredal 2023). This is maybe not surprising given humans’ predominantly anthropocentric viewpoint, but because of the sustainability context of our research, we ask ourselves if this prioritization of social issues and values may, in a complex manner, be a posteriorly located cause of our unsustainable development. Earlier empirical studies on human values, universal or not, seem to substantiate the social nature of values (Schwartz 1994; Hofstede 2011; Kenter et al. 2019). Schwartz (1994), for instance, identifies ten basic motivational values whom which all seem integral to ourselves and our sociality. Examples are power, achievement, hedonism and universalism—the latter encompassing considerations for both people and nature. Schwartz (1996, p. 121) also states that “values may play little role in behavior except when there is value conflict—when a behavior has consequences promotive of one (or more) value but opposed to others that are also cherished by the person”. In this way, he points exactly to the importance of values when a person needs to prioritize. Though acknowledging the cultural factors influencing the relationship between humans and nature (Archer 2009 [1996]; Burke et al. 2021), we find it interesting that Schwartz and Bardi (2001) also point to the fact that value hierarchies are strikingly similar across cultures. The potentially fundamental significance of our own research is also strengthened by new insights from neuroscience. Matthew Lieberman (2015), for instance, foreground the sociality of humankind by popularly calling it a super-power and challenges the psychologist Maslow's (1943) long-living and impactful hierarchy of needs, contending that our very existence hinges upon our social abilities and needs, nurtured from the inception of life.

O’Brien et al. (2023) commendably explore the understanding of human values within a broader context of sustainability transformations. The points made above, however, show that there is a potential oversight both on their part and within the wider research community. This oversight involves not fully considering the apparent connection of unsustainability with the evolution of our social priorities. Specifically, the prioritization of social values and issues across individual, group, political, and economic actions may have been insufficiently addressed. Drawing on insights from evolutionary theory, emerging neuroscience, and human value theories, we propose that the intricate workings of humans' social brains may be a root cause of unsustainable development. It could then also be suggested that the evolution of human sociality and social priorities over time align with what O’Brien et al. (2023) refer to as "universal values". Consequently, the structures and systems in the political sphere, as well as responses and behaviours in the practical sphere, would be significantly shaped by this social force. Therefore, integrating the inherent social preferences of humans into the foundational assumptions of the fractal approach to scaling sustainability transformations is crucial as it advances.

It is important to note that we do not claim to possess a definitive answer to the "why" question of unsustainable development. The term "social" might even seem simplistic in this context, and we acknowledge this limitation. In reality, the relationship between individuals' social priorities, unsustainable behaviour, and subsequent sustainability transformations is highly complex, extending beyond the boundaries of any single disciplinary domain or skill set. This complexity underscores the necessity for authentic interdisciplinary research, especially at the intersection of natural sciences such as neuroscience and evolutionary biology, in addition to psychology and social sciences. By delving into the nuanced connections among the functioning of our social brain, individual priorities and values, our comprehensive knowledge about unsustainable development, and the structural elements influencing opportunities, we advocate for an interdisciplinary approach to understand even more deeply why we act unsustainably. Consequently, enhancing our interdisciplinary understanding of the reasons behind unsustainable behaviour may contribute to further refine our understanding of how to approach sustainability transformation through the emergence of new fractal patterns.
